# Supplementary material for: Spectrally Selective Full-Color Single-Component Organic Photodetectors Based on Donor-Acceptor Conjugated Molecules
Source: Molecules. 2023 Jan 2;28(1):368. doi: 10.3390/molecules28010368 (PMC9823982; doi:10.3390/molecules28010368)
Supplement: Supplementary file 1 [file molecules-28-00368-s001.zip › molecules-2104589-supplementary.pdf]

Supporting information

Synthesis and characterization of materials

Materials

n-Butyllithium solution, 1.6 M in hexanes (*n*-BuLi), 2-ethylhexyl and 2-ethyl cyanoacetate, tetrakis(triphenylphosphine) palladium (0) ($Pd(PPh_3)_4$), heptanoyl chloride, tin tetrachloride were obtained from Sigma-Aldrich Co. and used without further purification. THF, pyridine, toluene and *N*,*N*-dimethylformamide (DMF) were dried and purified according to the known techniques.

5,5',5''-[Nitrilotris(4,1-phenylene)]trithiophene-2-carbaldehyde (**1**) was obtained as described in previous work [40]. 4-Bromo-7-(2-thienyl)-2,1,3-benzothiadiazole (**2**) [41] and 4,4′,4′′-tris(4,4,5,5-tetramethyl-1,3,2-dioxaborolan-2-yl)triphenylamine (**4**) [42] were obtained as reported elsewhere. All reactions, unless stated otherwise, were carried out under an inert atmosphere.

Synthesis

The elaborated synthetic route for the preparation of target materials **I** and **III** is shown on Scheme 1 of the manuscript, whereas the preparation of **II** and **IV** were described previously [32–34].

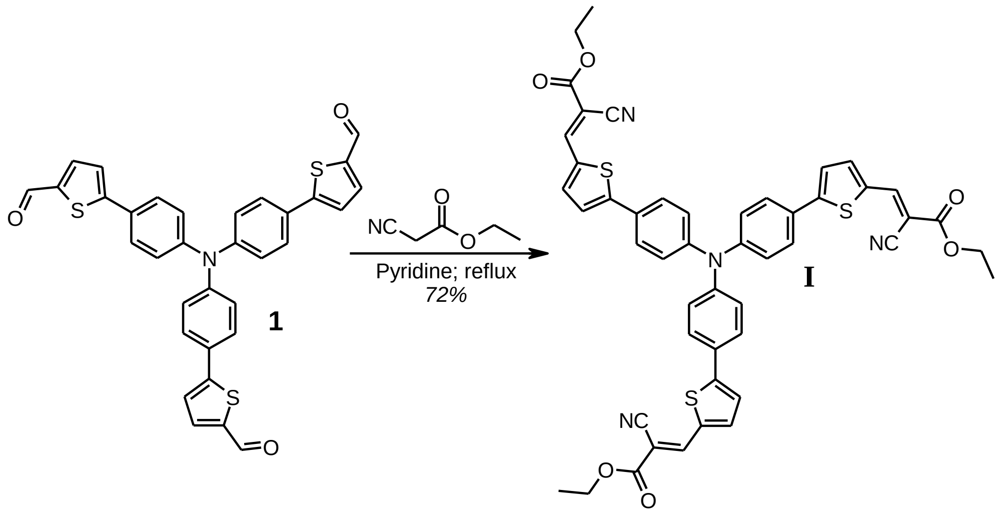

Triethyl(3,3',3''-((nitrilotris(benzene-4,1-diyl))tris(thiophene-5,2-diyl))(2*E*,2'*E*,2''*E*)-tris(2-cyanoacrylate) (I). Compound **1** (1.21g, 2.1 mmol), 2-ethyl cyanoacetate (1.07 g, 9.5 mmol) and dry pyridine (37 mL) were placed in a reaction vessel and stirred for 10 hours at reflux temperature using the microwave heating. After reaction was completed, pyridine was evaporated under vacuum and the residue solid was dried. The crude product was purified by column chromatography on silica gel using dichloromethane: ethyl acetate (20/1, *v*/*v*) as an eluent followed

by recrystallization from dichloromethane/hexane mixture to give pure **I** (1.29 g, 72%) as a red solid. *M.p.* = 288 °C. ^{1}H NMR (250 MHz, $CDCl_3$): 1.35–1.41 (t, 9H, *J* = 7.02 Hz); 4.31 (q, 6H, *J* = 7.02 Hz); 7.19 (d, 6H, *J* = 8.85 Hz); 7.38 (d, 3H, *J* = 3.97 Hz); 7.65 (d, 6H, *J* = 8.55 Hz); 7.74 (d, 3H, *J* = 3.97 Hz); 8.29 (s, 3H). ^{13}C NMR (75 MHz, $CDCl_3$): 14.08; 62.26; 98.37; 115.81; 123.92; 124.70; 127.77; 128.44; 134.81; 138.52; 145.95; 147.66; 153.69; 162.81. Calcd. (%) for $C_{48}H_{36}N_4O_6S_3$: C, 66.96; H, 4.21; N, 6.51; S, 11.17. Found: C, 67.06; H, 4.23; N, 6.49; S, 11.16.

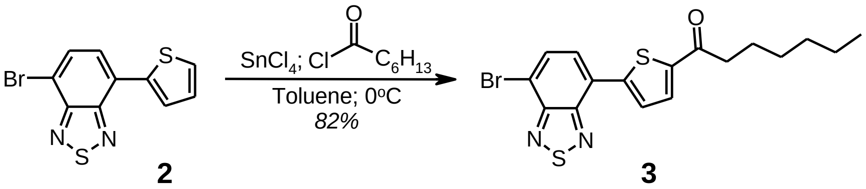

1-(5-(7-Bromobenzo[*c*][1,2,5]thiadiazol-4-yl)thiophen-2-yl)heptan-1-one) (3). $SnCl_4$ (14.79 g, 56.8 mmol) was added dropwise to the mixture of 4-bromo-7-(2-thienyl)-2,1,3-benzothiadiazole (14.34 g, 48.3 mmol) and heptanoyl chloride (7.89 g, 53.1 mmol) in 300 mL of dry toluene at 0 °C. The reaction mixture was stirred for 1 h at 0 °C, then the cooling bath was removed, and the stirring was continued for overnight with raising the temperature to RT. After reaction was completed, the reaction mixture was washed with 1M HCl, 300 mL H_2O, 5% Na_2CO_3. The combined organic phases were dried over sodium sulfate, filtered, evaporated and dried under vacuum. The crude product was purified by column chromatography on silica gel using toluene as an eluent followed by recrystallization from toluene:hexane (1/5, *v/v*) to give pure compound **3** (16.19 g, 82%) as a brown solid. *M.p.* = 141 °C. ^{1}H NMR (250 MHz, $CDCl_3$): 0.88 (t, 3H, *J* = 7.02 Hz); 1.22–1.53 (m, 6H); 1.76 (q, 2H, *J* = 7.32 Hz); 2.93 (t, 2H, *J* = 7.32 Hz); 7.76 (d, 1H, *J* = 3.97 Hz); 7.81 (d, 1H, *J* = 7.63 Hz); 7.90 (d, 1H, J = 7.63 Hz); 8.09 (d, 1H, *J* = 3.97 Hz). ^{3}C NMR (75 MHz, $CDCl_3$): 13.79; 22.36; 24.77; 28.94; 31.52; 39.41; 114.44; 126.25; 126.86; 128.44; 131.85; 132.04; 144.85; 145.48; 151.71; 153.91; 193.09. Calcd. (%) for $C_{17}H_{17}BrN_2OS_2$: C, 49.88; H, 4.19; N, 6.84; S, 15.67. Found: C, 49.97; H, 4.21; N, 6.88; S, 15.67.

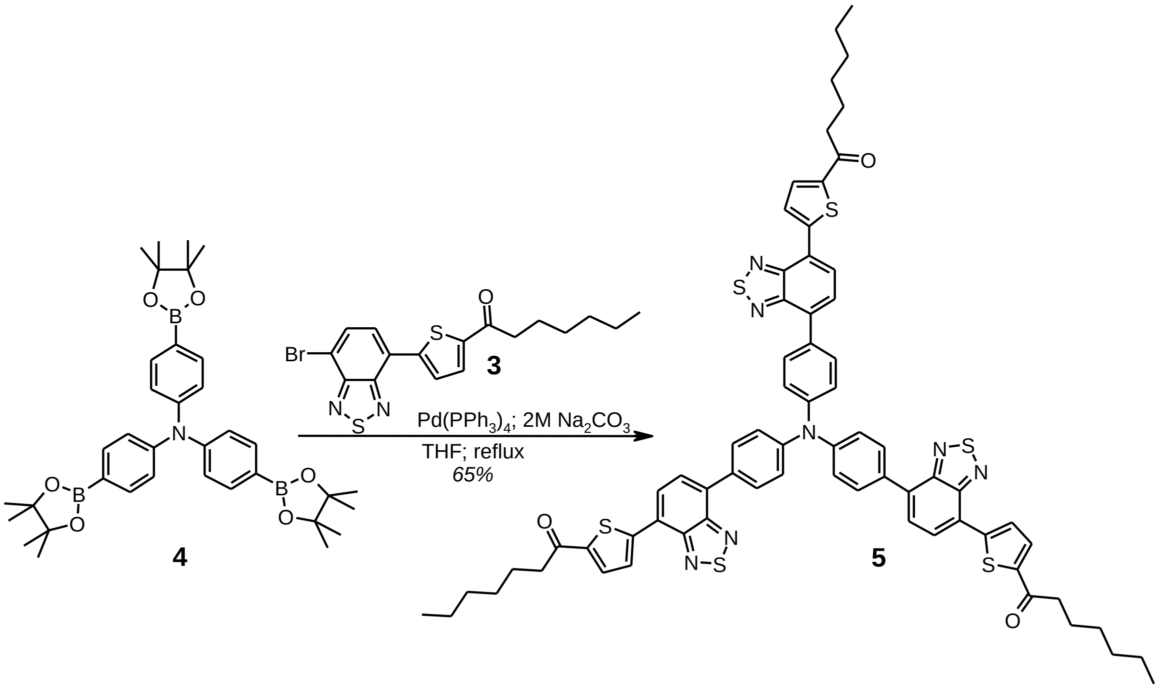

1,1',1''-(((nitrilotris(benzene-4,1-diyl))tris(benzo[*c*][1,2,5]thiadiazole-7,4-diyl))tris (thiophene-5,2-diyl))tris(heptan-1-one) (5). Compound **3** (3.38 g, 9.0 mmol), compound **4** (1.70 g, 3.0 mmol), $Pd(PPh_3)_4$ (0.24 g, 0.5 mmol), 2M solution of Na_2CO_3 (9 mL, 25 mmol) and THF (250 mL) were placed in a reaction vessel and stirred under inert atmosphere for 8 hours at reflux temperature. After reaction was completed, the reaction mixture was concentrated under reduced pressure. The crude product was purified by recrystallization from toluene to give pure compound **5** (2.19 g, 65%) as a red solid. *M.p.* = 127 ^{o}C. ^{1}H NMR (250 MHz, $CDCl_3$): 0.90 (t, 9H, J = 7.02 Hz); 1.29–1.46 (m, 18H); 1.79 (q, 6H, J = 7.02 Hz); 2.98 (t, 6H, J = 7.32 Hz); 7.44 (d, 6H, J = 8.55 Hz); 7.74–7.80 (m, 6H); 7.99 (d, 6H, J = 8.55 Hz); 8.05 (d, 3H, J = 7.32 Hz); 8.15 (d, 3H, J = 3.96 Hz). ^{13}C NMR (75 MHz, $CDCl_3$): 13.91; 22.45; 24.86; 29.02; 3159; 39.38; 124.42; 125.07; 127.06; 127.95; 130.34; 131.87; 132.06; 133.67; 144.20; 146.66; 147.51; 152.79; 153.85; 193.31. Calcd. (%) for $C_{69}H_{63}N_7O_3S_6$: C, 67.34; H, 5.16; N, 7.97; S, 15.63. Found: C, 67.39; H, 5.16; N, 7.95; S, 15.64.

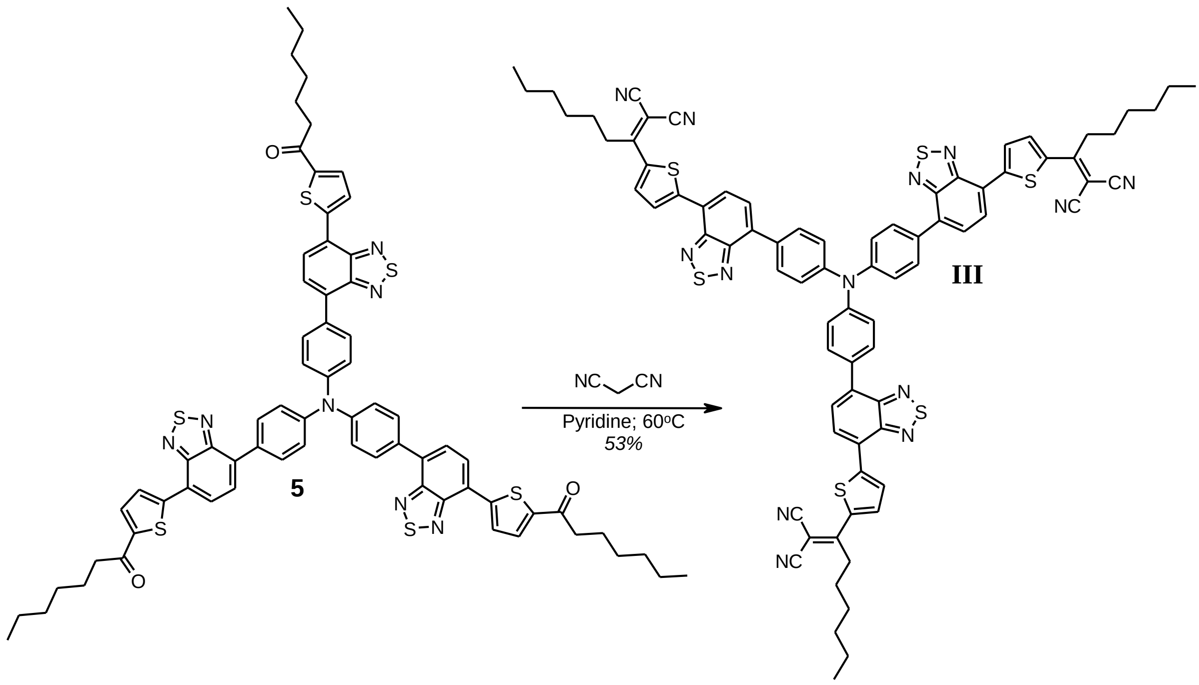

2,2',2''-((((nitrilotris(benzene-4,1-diyl))tris(benzo[*c*][1,2,5]thiadiazole-7,4-diyl))tris (thiophene-5,2-diyl))tris(heptan-1-yl-1-ylidene))trimalononitrile (III). Compound **5** (2.09 g, 1.7 mmol) and malononitrile (0.56 g, 8.5 mmol) were dissolved in pyridine (42 mL) in a round bottom flask under inert atmosphere. Then the mixture was stirred at 60°C for 8 h. After cooling to room temperature, the reaction mixture was concentrated under reduced pressure. The crude product was purified by column chromatography on silica gel using chloroform as an eluent, followed by recrystallization from toluene to give pure **III** (1.23 g, 53%) as a dark solid. *M.p.* = 257 °C. ^{1}H NMR (250 MHz, $CDCl_3$): 0.90 (t, 9H, J = 7.32 Hz); 1.29–1.40 (m, 12H); 1.43–1.54 (m, 6H); 1.76 (q, 6H, J = 7.63 Hz); 3.04 (t, 6H, J = 7.02 Hz); 7.46 (d, 6H, J = 8.54 Hz); 7.82 (d, 3H, J = 7.63 Hz); 8.03 (d, 6H, J = 8.54 Hz); 8.11 (d, 3H, J = 7.63 Hz); 8.14 (d, 3H, J = 4.28 Hz); 8.22 (d, 3H, J = 4.27 Hz). ^{13}C NMR (75 MHz, $CDCl_3$): 13.98; 22.40; 29.15; 30.38; 31.23; 37.58; 77.45; 113.71; 114.53; 123.75; 124.42; 126.99; 127.40; 128.38; 130.38; 131.56; 134.27; 134.35 137.61; 147.47; 147.99; 152.43; 153.59; 167.04. Calcd. (%) for $C_{78}H_{63}N_{13}S_6$: C, 68.14; H, 4.62; N, 13.24; S, 13.99. Found: C, 68.21; H, 4.65; N, 13.20; S, 13.96. MALDI-TOF MS: found m/z 1372.76; calcd. for $[M]^+$ 1373.37.

The Knövenagel condensation was carried out in the microwave “Discovery”, (CEM corporation, USA), using a standard method with the open vessel option, 30 watts.

Characterization

NMR spectra. ^{1}H NMR spectra were recorded using a "Bruker WP-250 SY" spectrometer, working at a frequency of 250 MHz and using $CDCl_3$ (7.25 ppm) signals as the internal standard. ^{13}C NMR spectra were recorded using a "Bruker Avance II 300" spectrometer, working at a frequency of 75 MHz. In the case of ^{1}H NMR spectroscopy, the compounds to be analyzed were taken in the form of 1% solutions in $CDCl_3$. In the case of ^{13}C NMR spectroscopy, the compounds to be analyzed were taken in the form of 5% solutions in $CDCl_3$. The spectra were then processed on the computer using the "ACD Labs" software.

Elemental analysis. Elemental analysis of C, N and H elements was carried out using CHN automatic analyzer "CE 1106" (Italy). The settling titration using $BaCl_2$ was applied to analyze the S element.

Mass-spectra. Mass-spectra (MALDI-TOF) were registered on an "Autoflex II Bruker" (resolution FWHM 18000), equipped with a nitrogen laser (work wavelength 337 nm) and time-of-flight mass-detector working in the reflections mode. The accelerating voltage was 20 kV. Samples were applied to a polished stainless-steel substrate. Spectrum was recorded in the positive ion mode. The resulting spectrum was the sum of 300 spectra obtained at different points of the sample. 2,5-Dihydroxybenzoic acid (DHB) (Acros, 99%) and α-cyano-4-hydroxycinnamic acid (HCCA) (Acros, 99%) were used as matrices.

Absorption spectra. The absorption spectra were recorded on a Shimadzu UV-2501PC (Japan) spectrophotometer in the standard 10 mm photometric quartz cuvette using THF solutions with the concentrations of 10^{-5} M. Films were cast from THF solutions on glass substrates. All measurements were carried out at room temperatures.

^{1}H and ^{13}C NMR Spectra

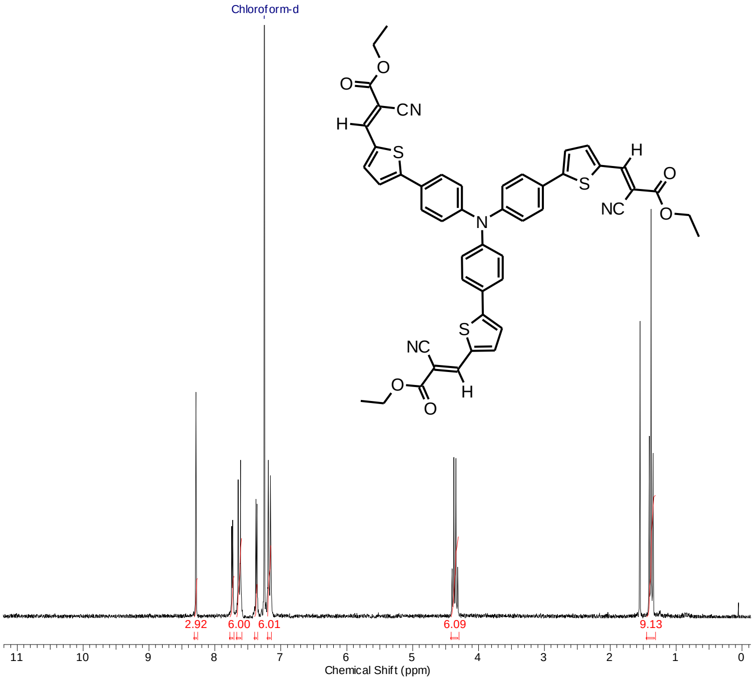

No.	Annotation	(ppm)
1	Chloroform-d	7.25

No.	(ppm)	Value	Absolute Value
1	[1.31 .. 1.45]	9.125	1.56998e+7
2	[4.30 .. 4.41]	6.088	1.04734e+7
3	[7.14 .. 7.20]	6.014	1.03461e+7
4	[7.35 .. 7.40]	2.878	4.95097e+6
5	[7.59 .. 7.67]	6.000	1.03228e+7
6	[7.71 .. 7.77]	3.099	5.33099e+6
7	[8.26 .. 8.32]	2.920	5.02293e+6

Figure S1. ^{1}H NMR spectrum of target material **I**.

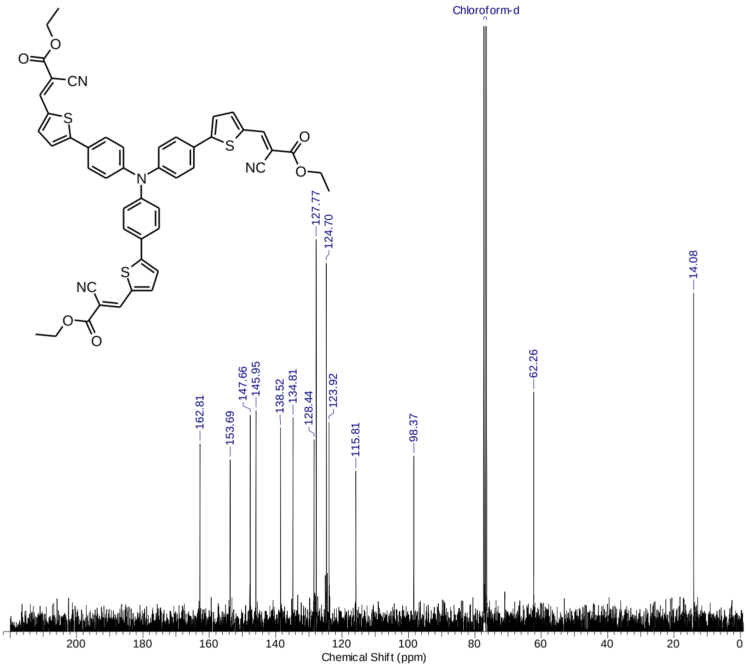

No.	(ppm)	(Hz)	Height
1	14.08	1062.7	0.3323
2	62.26	4699.5	0.2359
3	98.37	7424.6	0.1733
4	115.81	8741.0	0.1589
5	123.92	9353.6	0.2062
6	124.70	9412.2	0.3613
7	127.77	9643.8	0.3844
8	128.44	9694.7	0.1894
9	134.81	10175.1	0.2109
10	138.52	10454.9	0.2010
11	145.95	11016.1	0.2177
12	147.66	11144.9	0.2132
13	153.69	11600.4	0.1698
14	162.81	12288.8	0.1855

No.	Annotation	(ppm)
1	Chloroform-d	[76.47 .. 77.30]

Figure S2. ^{13}C NMR spectrum of target material **I**.

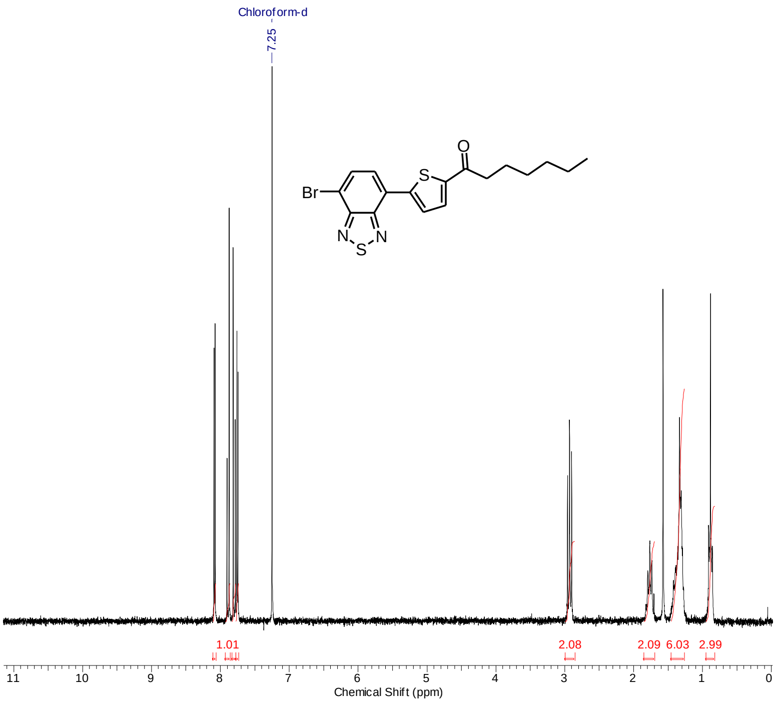

No.	Annotation	(ppm)
1	Chloroform-d	7.25

No.	(ppm)	Value	Absolute Value
1	[0.82 .. 0.95]	2.992	1.71125e+7
2	[1.26 .. 1.46]	6.027	3.44743e+7
3	[1.69 .. 1.85]	2.087	1.19389e+7
4	[2.85 .. 3.00]	2.085	1.19227e+7
5	[7.73 .. 7.77]	1.002	5.73113e+6
6	[7.77 .. 7.83]	0.995	5.69174e+6
7	[7.86 .. 7.93]	1.007	5.76094e+6
8	[8.06 .. 8.11]	1.000	5.71961e+6

Figure S3. ^{1}H NMR spectrum of compound **3**.

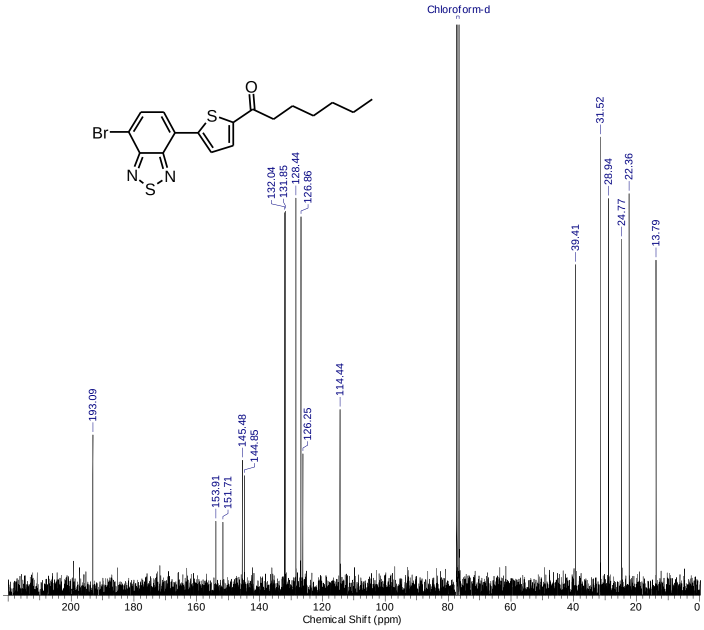

No.	(ppm)	(Hz)	Height
1	13.79	1040.6	0.4488
2	22.36	1688.0	0.5375
3	24.77	1869.9	0.4766
4	28.94	2184.5	0.5311
5	31.52	2379.1	0.6129
6	39.41	2974.5	0.4427
7	114.44	8637.6	0.2492
8	126.25	9529.4	0.1897
9	126.86	9575.3	0.5064
10	128.44	9694.1	0.5316
11	131.85	9951.8	0.5141
12	132.04	9966.2	0.5123
13	144.85	10933.1	0.1604
14	145.48	10980.7	0.1809
15	151.71	11450.6	0.0981
16	153.91	11616.5	0.0994
17	193.09	14574.3	0.2150

No.	Annotation	(ppm)
1	Chloroform-d	[76.47 .. 77.32]

Figure S4. ^{13}C NMR spectrum of compound **3**.

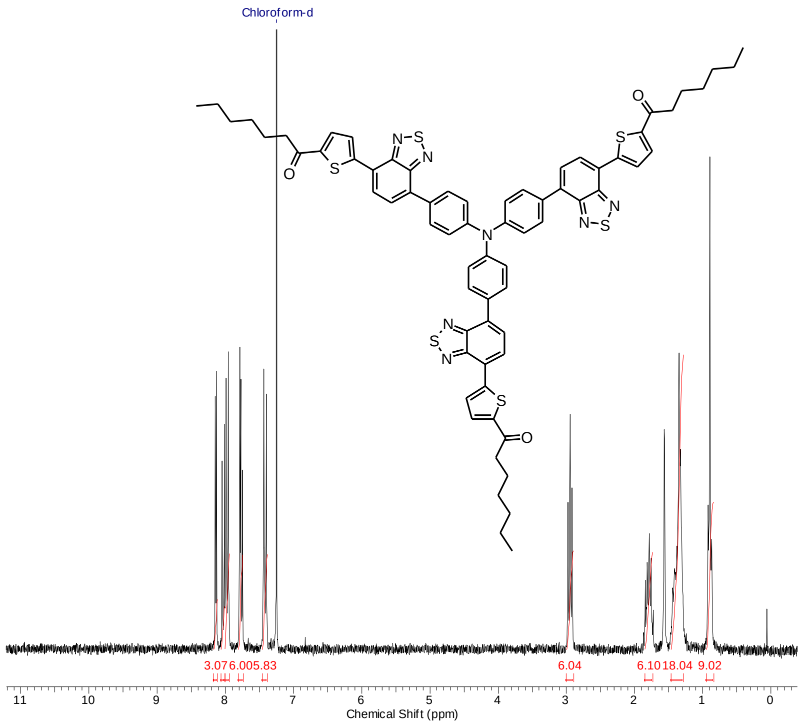

No.	Annotation	(ppm)
1	Chloroform-d	7.25

No.	(ppm)	Value	Absolute Value
1	[0.84 .. 0.95]	9.020	2.07035e+7
2	[1.28 .. 1.47]	18.036	4.13985e+7
3	[1.73 .. 1.85]	6.099	1.39993e+7
4	[2.89 .. 3.00]	6.038	1.38600e+7
5	[7.38 .. 7.46]	5.833	1.33884e+7
6	[7.74 .. 7.81]	6.000	1.37718e+7
7	[7.94 .. 8.01]	5.982	1.37297e+7
8	[8.01 .. 8.06]	3.149	7.22869e+6
9	[8.11 .. 8.17]	3.069	7.04348e+6

Figure S5. ^{1}H NMR spectrum of compound **5**.

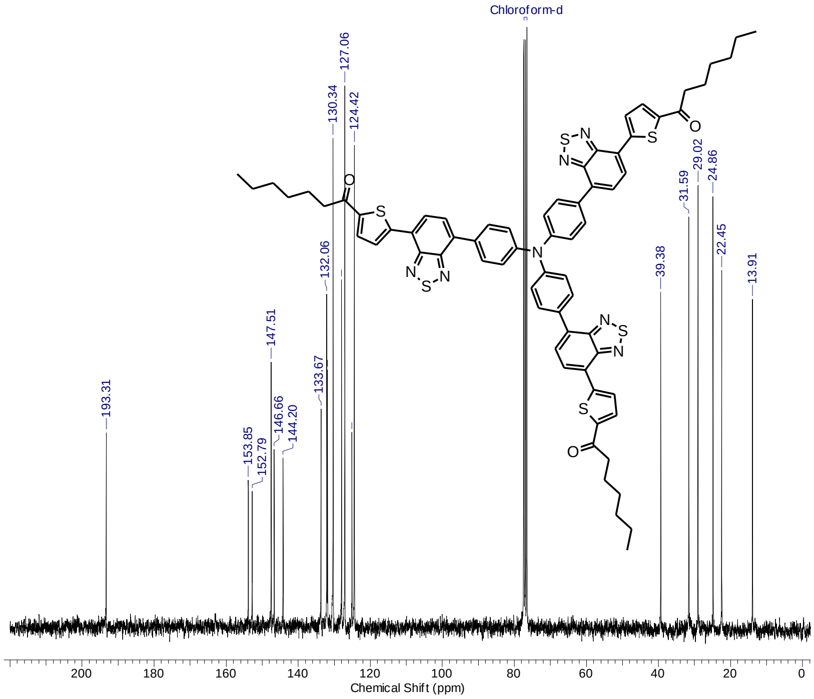

No.	(ppm)	(Hz)	Height
1	13.91	1050.0	0.5555
2	22.45	1694.6	0.6028
3	24.86	1876.5	0.7232
4	29.02	2190.6	0.7416
5	31.59	2384.6	0.6898
6	39.38	2972.3	0.5669
7	124.42	9391.2	0.8072
8	125.07	9440.4	0.3386
9	127.06	9590.2	0.9038
10	127.95	9657.7	0.5878

No.	(ppm)	(Hz)	Height
11	130.34	9837.9	0.8183
12	131.87	9952.9	0.4404
13	132.06	9967.8	0.5641
14	133.67	10088.9	0.3767
15	144.20	10883.9	0.2967
16	146.66	11069.7	0.3106
17	147.51	11133.8	0.4533
18	152.79	11531.9	0.2420
19	153.85	11612.1	0.2604
20	193.31	14590.4	0.3377

No.	Annotation	(ppm)
1	Chloroform-d	[76.53 .. 77.37]

Figure S6. ^{13}C NMR spectrum of compound **5**.

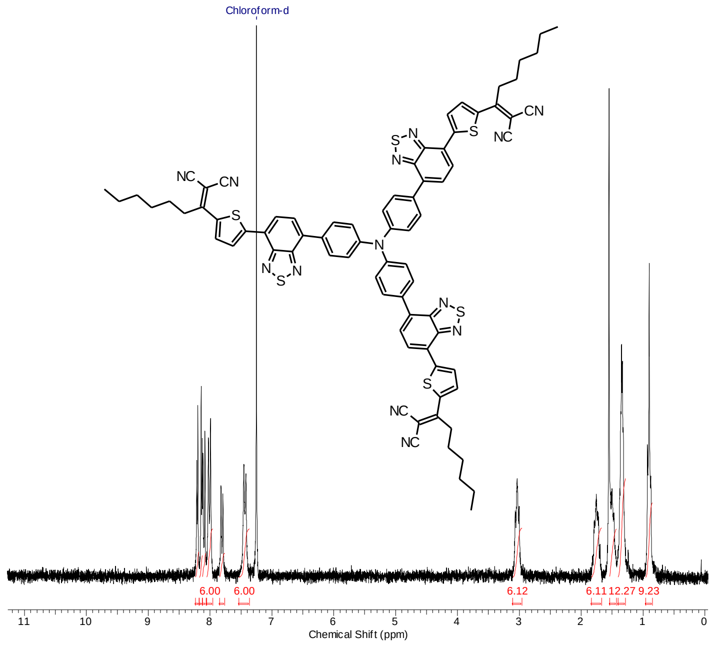

No.	Annotation	(ppm)
1	Chloroform-d	7.25

No.	(ppm)	Value	Absolute Value
1	[0.85 .. 0.96]	9.234	2.98972e+7
2	[1.28 .. 1.40]	12.271	3.97296e+7
3	[1.43 .. 1.54]	6.026	1.95108e+7
4	[1.67 .. 1.84]	6.115	1.97977e+7
5	[2.96 .. 3.11]	6.124	1.98275e+7
6	[7.36 .. 7.53]	6.000	1.94265e+7
7	[7.76 .. 7.85]	3.005	9.73009e+6
8	[7.95 .. 8.05]	6.001	1.94311e+7
9	[8.07 .. 8.12]	3.037	9.83267e+6
10	[8.13 .. 8.17]	3.029	9.80713e+6
11	[8.18 .. 8.24]	3.188	1.03226e+7

Figure S7. ^{1}H NMR spectrum of target material **III**.

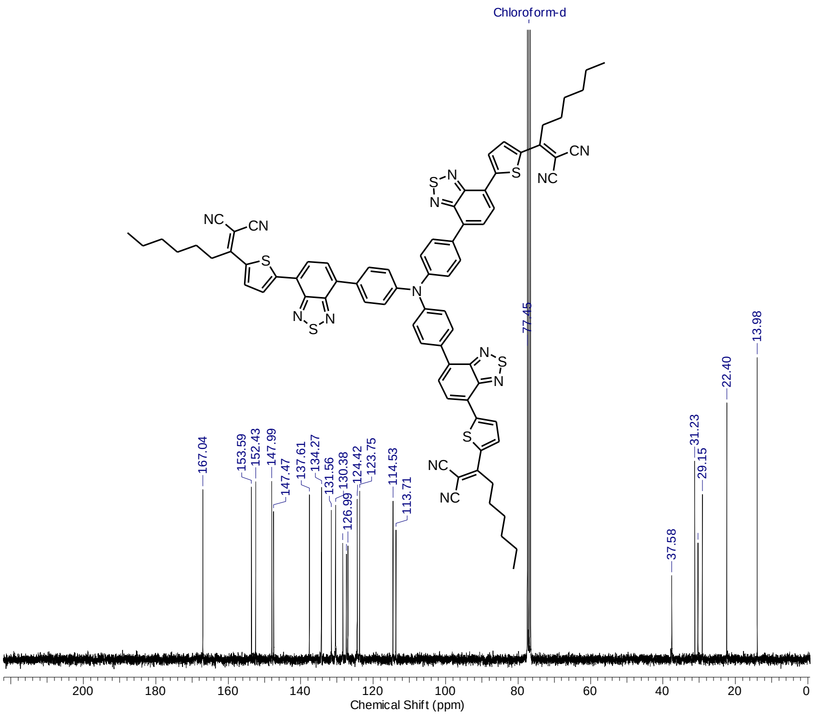

No.	(ppm)	(Hz)	Height
1	13.98	1054.9	0.3220
2	22.40	1690.7	0.2739
3	29.15	2199.9	0.1759
4	30.38	2292.8	0.1242
5	31.23	2357.5	0.2117
6	37.58	2836.2	0.0893
7	77.45	5846.1	0.3310
8	113.71	8582.2	0.1382
9	114.53	8644.2	0.1689
10	123.75	9340.2	0.1794
11	124.42	9391.1	0.1710
12	126.99	9584.6	0.1212

No.	(ppm)	(Hz)	Height
13	127.40	9616.1	0.1124
14	128.38	9689.6	0.1237
15	130.38	9840.6	0.1644
16	131.56	9929.6	0.1589
17	134.27	10134.1	0.1837
18	134.35	10140.2	0.1140
19	137.61	10386.8	0.1755
20	147.47	11130.4	0.1578
21	147.99	11170.2	0.1899
22	152.43	11504.7	0.1896
23	153.59	11592.6	0.1839
24	167.04	12607.7	0.1811

No.	Annotation	(ppm)
1	Chloroform-d	77.00

Figure S8. ^{13}C NMR spectrum of target material **III**.

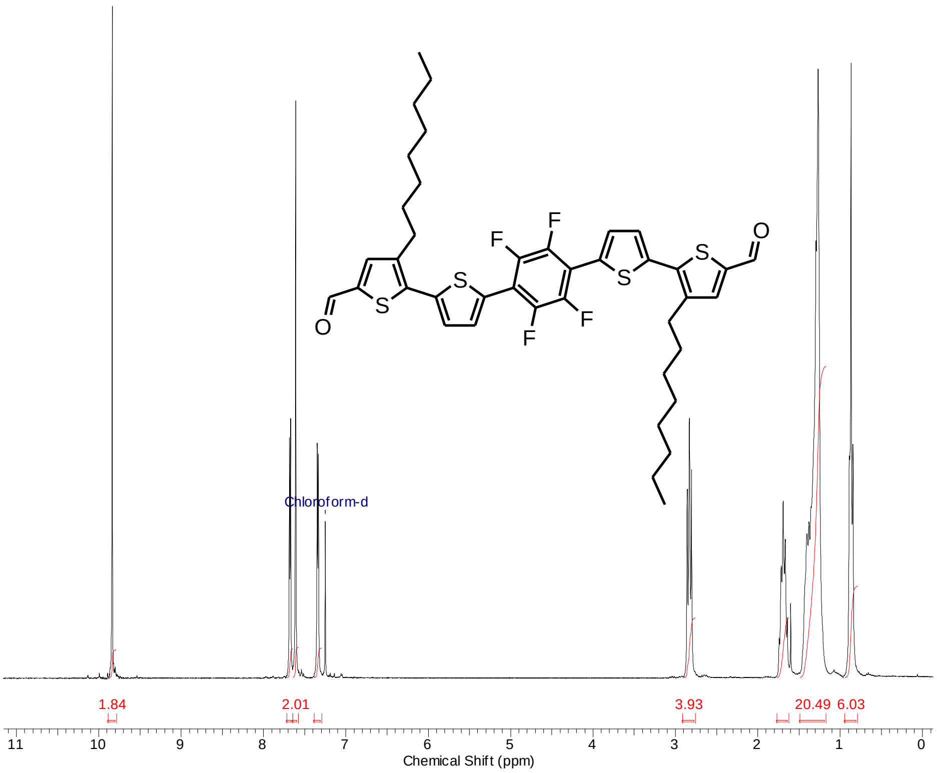

No.	Annotation	(ppm)
1	Chloroform-d	7.25

No.	(ppm)	Value	Absolute Value
1	[0.79 .. 0.94]	6.034	7.94622e+9
2	[1.17 .. 1.49]	20.494	2.69876e+10
3	[1.62 .. 1.77]	3.959	5.21290e+9
4	[2.76 .. 2.91]	3.932	5.17792e+9
5	[7.29 .. 7.39]	1.959	2.58012e+9
6	[7.58 .. 7.64]	2.012	2.64899e+9
7	[7.65 .. 7.72]	1.891	2.49035e+9
8	[9.78 .. 9.89]	1.839	2.42164e+9

Figure S9. ^{1}H NMR spectrum of target material **IV**.

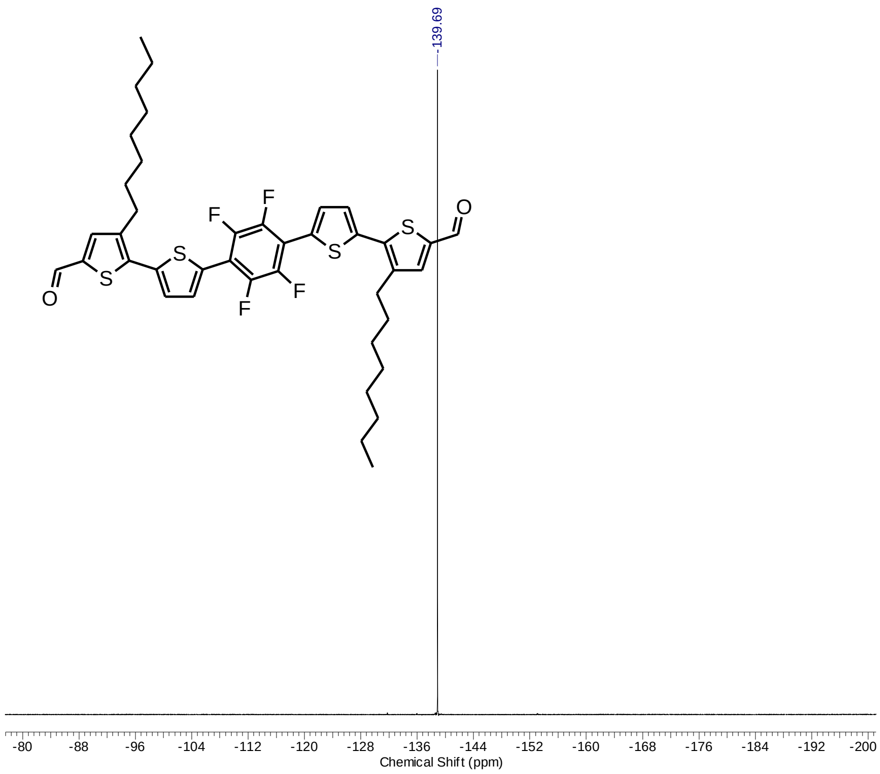

No.	(ppm)	(Hz)	Height
1	-139.69	-39453.7	1.0000

Figure S10. ^{19}F NMR (282 MHz, $CDCl_3$) spectrum of target material **IV**.

Optical properties

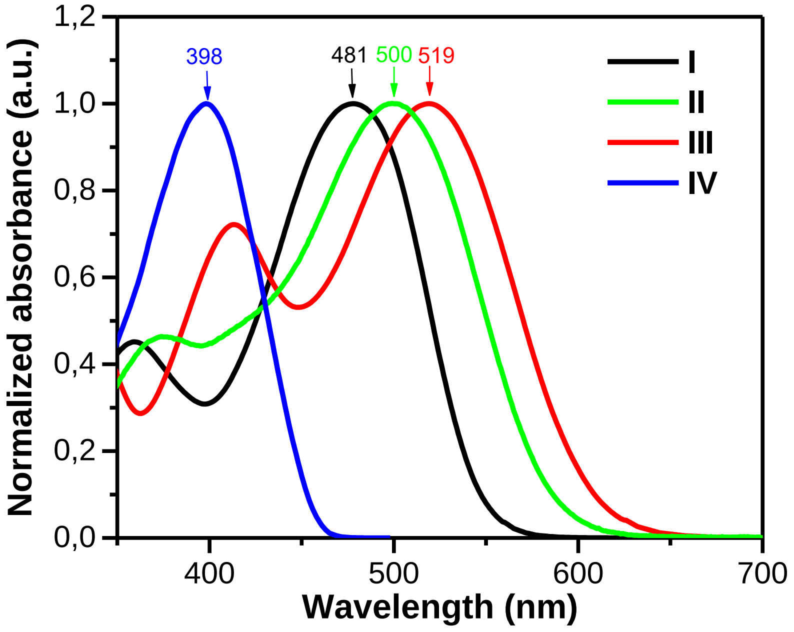

Figure S11. UV-vis normalized absorption spectra of materials studied in THF solution.

Mobility measurements

Hole mobility in films

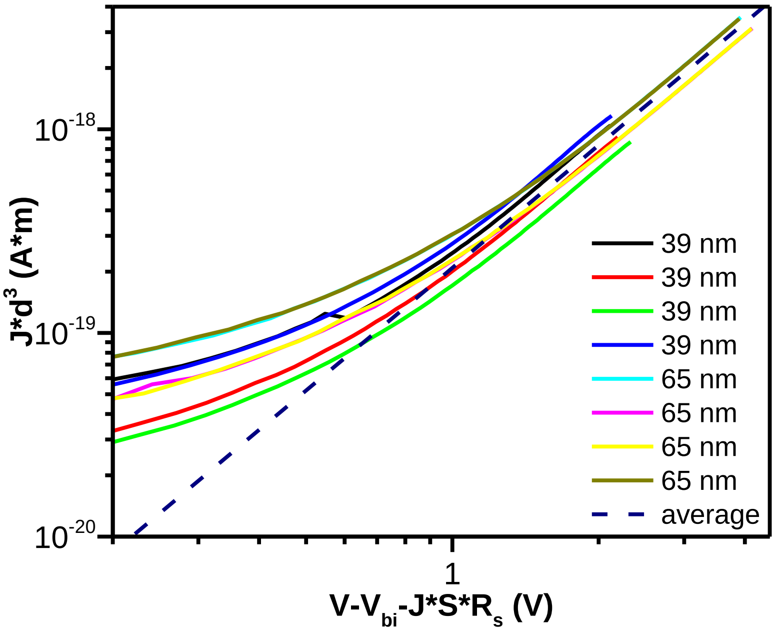

Figure S12. *J–V* characteristics of **I** hole only devices (solid lines). Jd^3 is plotted for two different layer thicknesses (39±3 and 65±3 nm) against the voltage, corrected for the built-in voltage and the electrode series resistance. Dashed line represents SCLC-fit, from which the hole mobility is extracted.

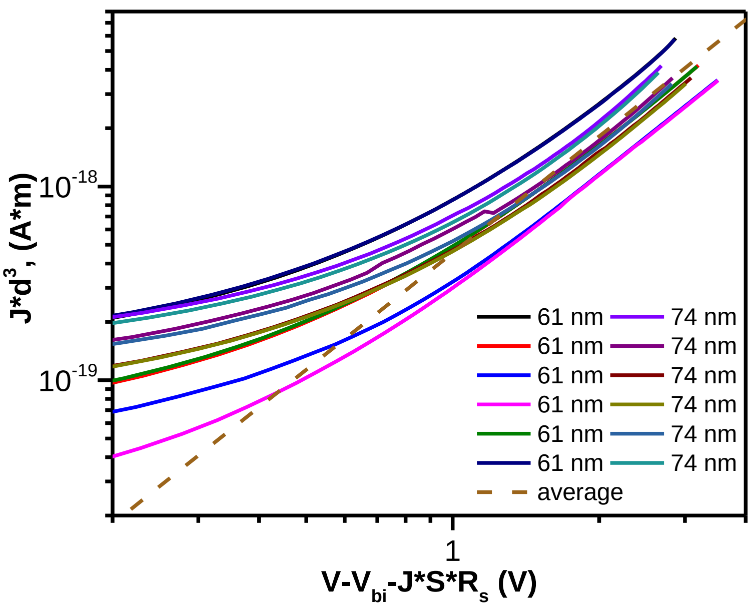

Figure S13. *J–V* characteristics of **II** hole only devices (solid lines). Jd^3 is plotted for two different layer thicknesses (61±3 and 74±3 nm) against the voltage, corrected for the built-in voltage and the electrode series resistance. Dashed line represents SCLC-fit, from which the hole mobility is extracted.

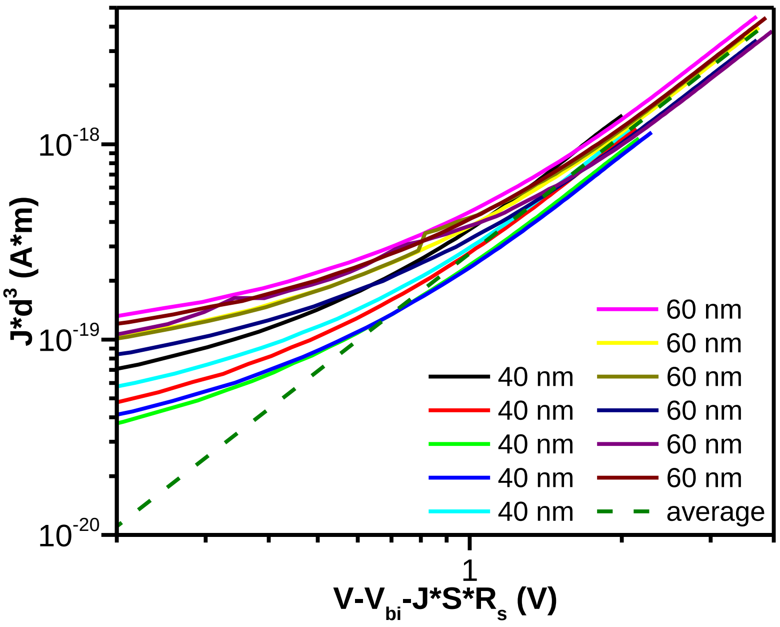

Figure S14. *J–V* characteristics of **III** hole only devices (solid lines). Jd^3 is plotted for two different layer thicknesses (40±3 and 60±3 nm) against the voltage, corrected for the built-in voltage and the electrode series resistance. Dashed line represents SCLC-fit, from which the hole mobility is extracted.

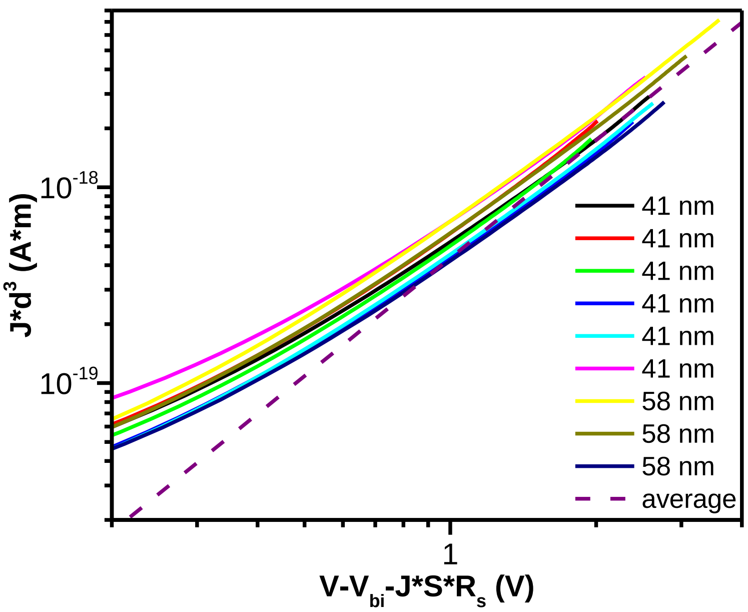

Figure S15. *J–V* characteristics of **IV+PC$_{71}$BM(10%)** hole only devices (solid lines). *Jd*^{3} is plotted for two different layer thicknesses (41±3 and 58±3 nm) against the voltage, corrected for the built-in voltage and the electrode series resistance. Dashed line represents SCLC-fit, from which the hole mobility is extracted.

Electron mobility in films

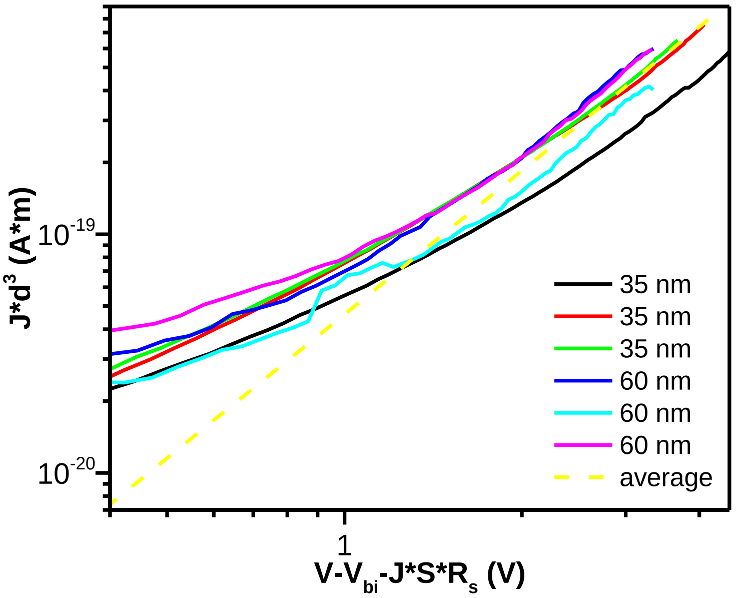

Figure S16. *J–V* characteristics of **I** electron only devices (solid lines). *Jd*^{3} is plotted for two different layer thicknesses (35±3 and 60±3 nm) against the voltage, corrected for the built-in voltage and the electrode series resistance. Dashed line represents SCLC-fit, from which the electron mobility is extracted.

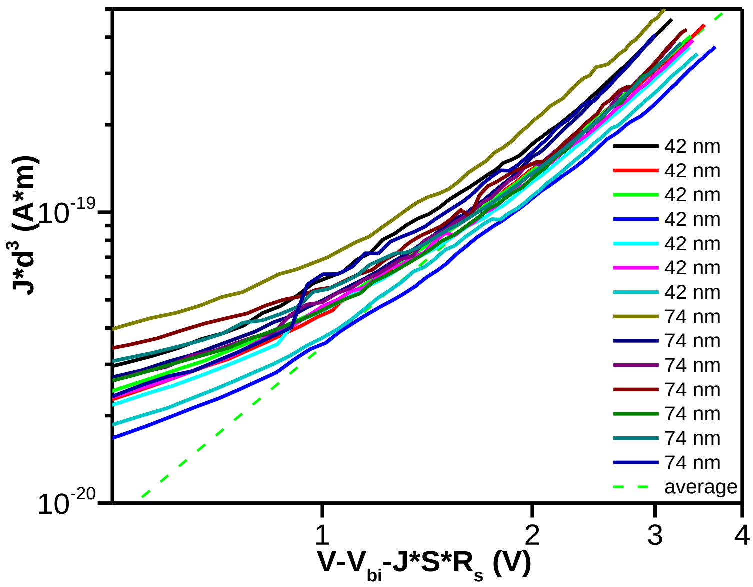

Figure S17. *J*–*V* characteristics of **II** electron only devices (solid lines). Jd^3 is plotted for two different layer thicknesses (42±3 and 74±3 nm) against the voltage, corrected for the built-in voltage and the electrode series resistance. Dashed line represents SCLC-fit, from which the electron mobility is extracted.

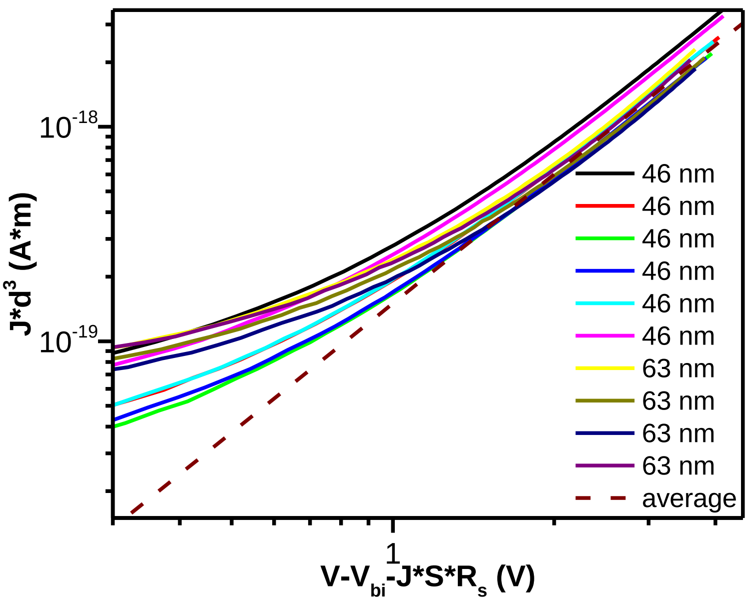

Figure S18. *J*–*V* characteristics of **III** electron only devices (solid lines). Jd^3 is plotted for two different layer thicknesses (46±3 and 63±3 nm) against the voltage, corrected for the built-in voltage and the electrode series resistance. Dashed line represents SCLC-fit, from which the electron mobility is extracted.

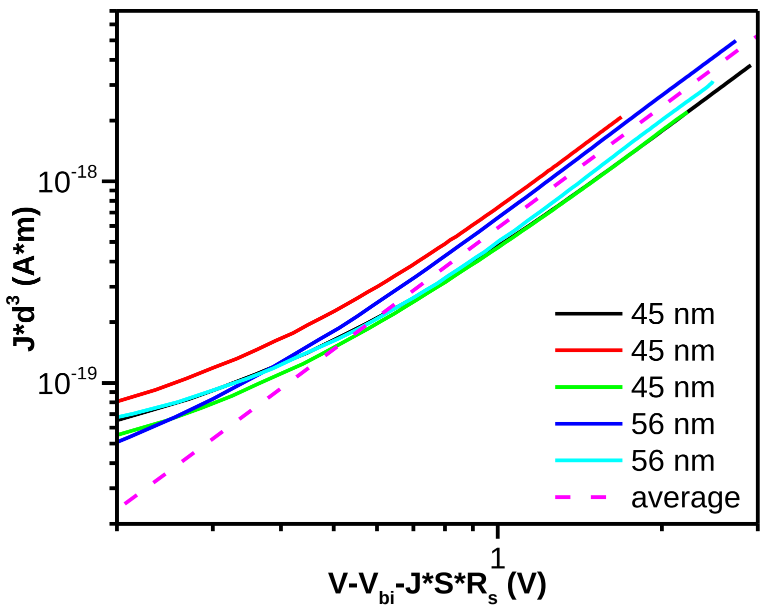

Figure S19. *J*–*V* characteristics of **IV+PC$_{71}$BM(10%)** electron only devices (solid lines). Jd^3 is plotted for two different layer thicknesses (45±3 and 56±3 nm) against the voltage, corrected for the built-in voltage and the electrode series resistance. Dashed line represents SCLC-fit, from which the electron mobility is extracted.

Photodetectors J-V curves, photovoltaic parameters, and EQE spectra.

The main photovoltaic properties of single-component photodetectors are presented in Figure S20 and summarized in Table S1. EQE and normalized EQE spectra are presented in Figure S21. Maximum EQE values and positions of peaks are summarized in Table S2.

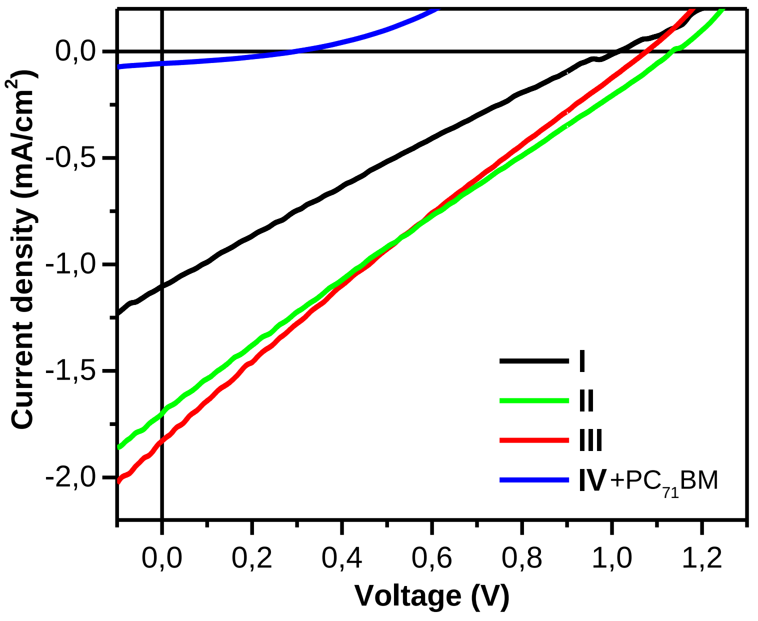

Figure S20. *J-V* curves of photodetectors under AM1.5G illumination (100 mW/cm^2) of solar simulator (Newport 67005).

Table S1. The best photovoltaic parameters of single-component photodetectors under AM1.5G illumination (100 mW/cm^2).

Active layer material	J_{SC}, mA/cm^2	$J_{SC\ EQE}$*, mA/cm^2	V_{OC}, V	FF, %	PCE, %
**I**	1.10	0.86	1.01	23.4	0.26
**II**	1.70	1.55	1.13	24.3	0.47
**III**	1.83	1.67	1.08	23.6	0.47
**IV+PC$_{71}$BM(10%)**	0.057	0.043	0.30	32.8	0.0055

Note: *$J_{SC\ EQE}$ is the short-circuit current integrated from the EQE spectrum.

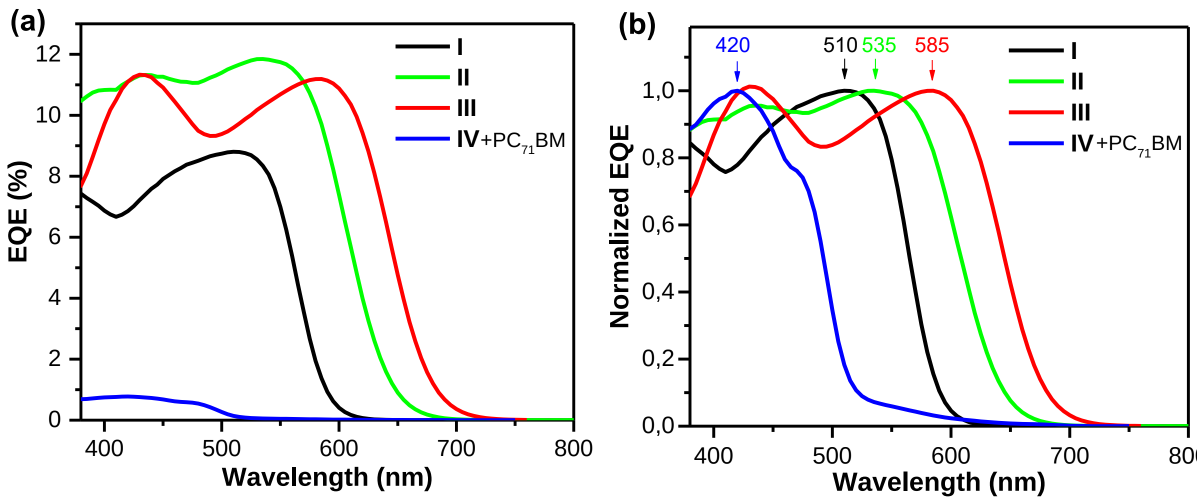

Figure S21. EQE (a) and normalized EQE (b) spectra of photodetectors. Arrows with numbers indicate the maxima of the spectra and their position.

Table S2. Maximum EQE values and positions of peaks.

Photoactive material	EQE, %	λ_{max}^{EQE}, nm
**I**	8.8	510
**II**	12	535
**III**	11	585
**IV+PC$_{71}$BM(10%)**	0.77	420
